# Analysis at the single-cell level indicates an important role of heterogeneous global DNA methylation status on the progression of lung adenocarcinoma

**DOI:** 10.1038/s41598-021-02786-y

**Published:** 2021-12-02

**Authors:** Quan-Fang Chen, Han Gao, Qing-Yun Pan, Ying-Ju Wang, Xiao-Ning Zhong

**Affiliations:** Department of Respiratory, The First Affiliated Hospital, Guangxi Medical University, 6 Shuangyong Road, Nanning, 530021 Guangxi People’s Republic of China

**Keywords:** Cancer therapy, Lung cancer, Cancer, Cell biology

## Abstract

Aberrant DNA modifications affect the tumorigenesis and progression of lung cancer. However, the global methylation status of tumor cells and the heterogeneous methylation status of cells within the same tumor need further study. We used publicly available single-cell RNAseq data to investigate the impact and diversity of global methylation status on lung adenocarcinoma. Clustering cells into subgroups and cell differentiation pseudotime analysis, based on expression profile, demonstrated that the global methylation status was crucial to lung adenocarcinoma function and progression. Hypermethylated tumor cells had increased activity related to the hypoxia response. Hyper- and hypomethylated cells indicated upregulation in pathways involving focal adhesion and cell junctions. Pseudotime analysis showed that cell clusters with unique methylation activities were located at the ends of the putative trajectories, suggesting that DNA methylation and demethylation activities were essential to tumor cell progression. Expression of *SPP1* was associated with the global methylation status of tumor cells and with patient prognosis. Our study identified the importance and diversity of global DNA methylation status by analysis at the single-cell level. Our findings provide new information about the global DNA methylation status of tumor cells and suggest new approaches for precision medical treatments for lung adenocarcinoma.

## Introduction

Lung cancer is a commonly diagnosed cancer that is a major cause of cancer deaths worldwide. Although deaths from lung cancer have declined dramatically during recent years, it is still the second most commonly diagnosed cancer and is responsible for most cancer deaths in both sexes^1^.

Genetic alterations are common in cancer, and DNA methylation is a common epigenetic modification present in the CpG-rich islands of cancer patients^2^. In particular, cancer tissues often have hypermethylation in the promoter regions of tumor suppressor genes, but hypomethylation of the genome overall^3^. DNA methylation patterns are associated with different gene expression profiles. Previous studies of the biological role of DNA methylation in lung cancer mainly focused on single genes, such as the relationship of the methylation of a promoter of a specific gene with its expression, and the relationship of the expression of this specific gene with oncogenesis and tumor progression. For example, previous studies reported hyper-methylation of the promoters of *CDKN2A*, *CDH13*, and *APC* in lung cancer^4^. Despite extensive research on the hypermethylation of gene promoters, few studies have examined the genome-wide methylation activity of lung cancer cells. Tumors consist of cells with diverse molecular signatures, and this heterogeneity increases as cancer progresses^5^. The development of single-cell sequencing technology made it possible to profile global methylation levels in lung cancer at the resolution of single cells.

In this study, we used single-cell sequencing data from 3 patients with lung adenocarcinomas, and compared expression profiles of samples from the core, middle, and border of the tumor. We assessed the global methylation level using gene set variation analysis (GSVA) score on gene ontology (GO) items (GO_DNA_METHYLATION and GO_DNA_DEMETHYLATION) and then compared the overall methylation level of each cell cluster to identify hyper- and hypo-methylation profiles. We also used single-cell pseudotime analysis to verify the role of global methylation on the progression of lung adenocarcinoma.

## Results

### Subtyping of lung adenocarcinoma tumor cells

**Figure 1:**
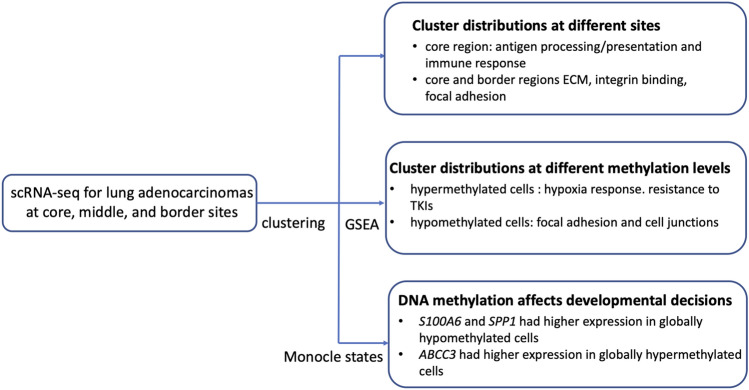
Flowchart showing the analysis pipeline and main findings.

**Figure 2:**
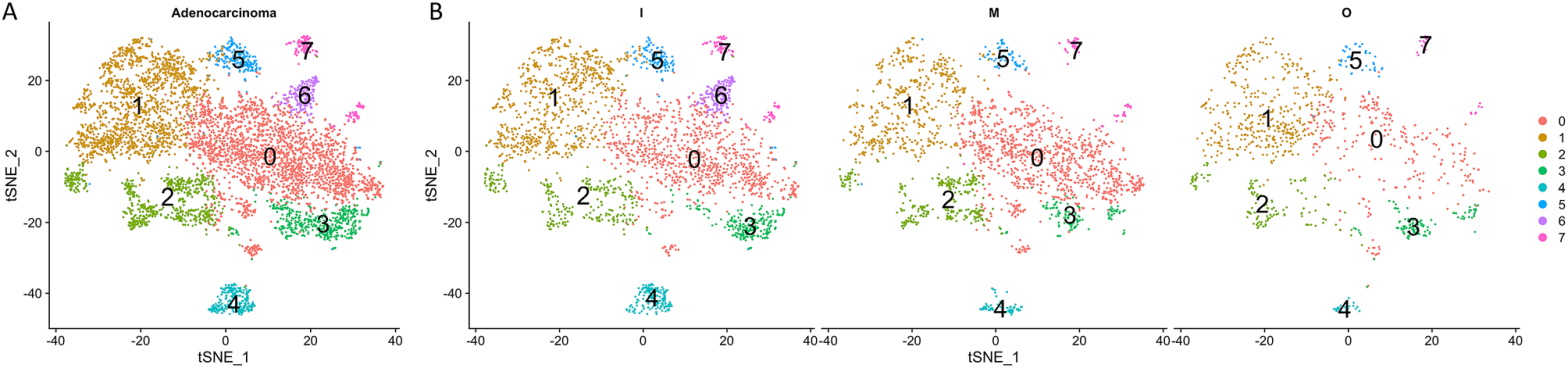
t-distributed stochastic neighbor embedding plots of the eight clusters from Seurat analysis (**A**) and of the eight clusters according to tumor site (**B**). *I* inner (core), *M* middle, *O* outer (border).

Tumor heterogeneity refers to the presence cells within the same tumor that differ in morphology and other phenotypic characteristics. We used the Seurat pipeline (Fig. 1) to characterize the detailed DNA methylation status of 6251 tumor cells from 3 patients with lung adenocarcinomas at the core, middle, and border sites into eight clusters (Fig. 2A). Analysis of these data using t-SNE plots according to sample site showed that cells from cluster-6 were all from the core region of the tumor, cells from cluster-4 were enriched in the core region, and cells from cluster-5 were depleted from the middle region (Fig. 2B).

**Figure 3:**
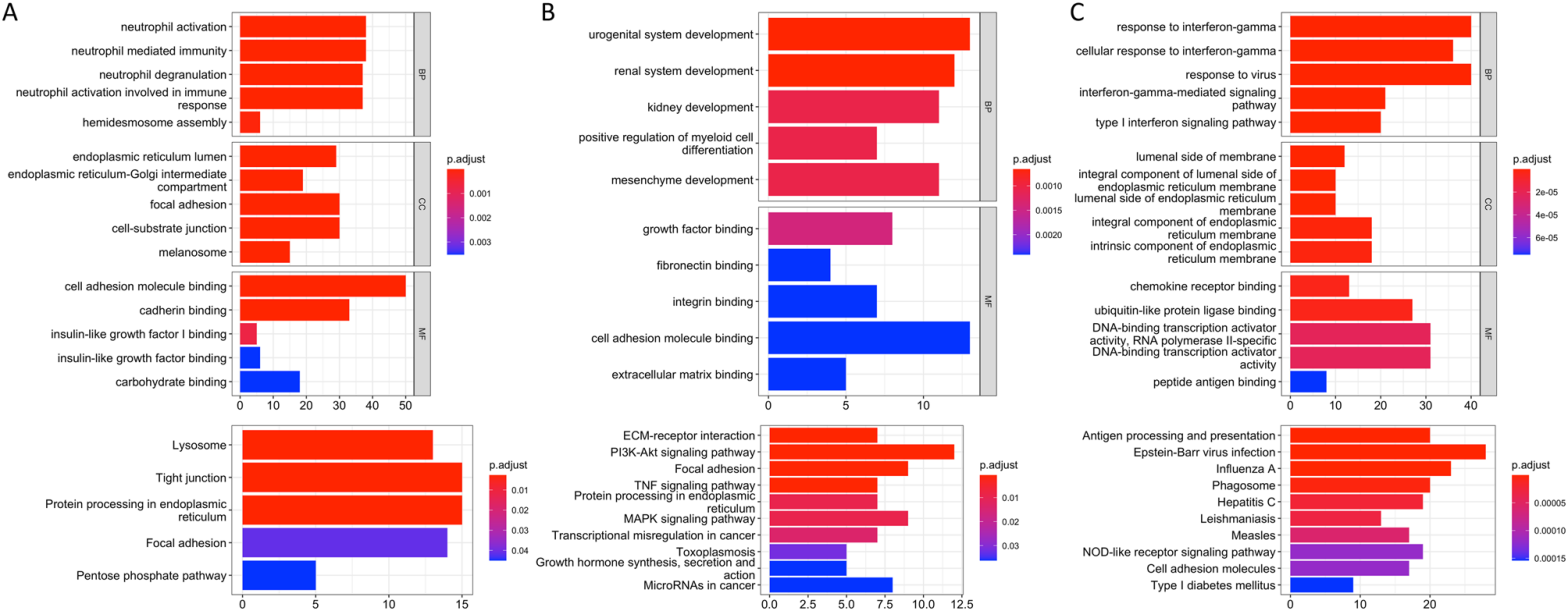
Kyoto Encyclopedia of Genes and Genomes and Gene Ontology enrichment analysis of Cluster-4 (**A**), Cluster-5 (**B**), and Cluster-6 (**C**). *BP* biological process, *CC* cellular components, *MF* molecular functions.

GO and KEGG enrichment analysis for genes highly expressed in each cluster demonstrated that cells at the tumor core were enriched in functions related to antigen processing/presentation and immune response (Cluster-4 in Fig. 3A and Cluster-6 in Fig. 3C). In addition, cells at the core and border regions had higher activities in extracellular matrix (ECM), integrin binding, and focal adhesion (Cluster-5 in Fig. 3B).

### DNA methylation and de-methylation activity in different tumor subtypes

**Figure 4:**
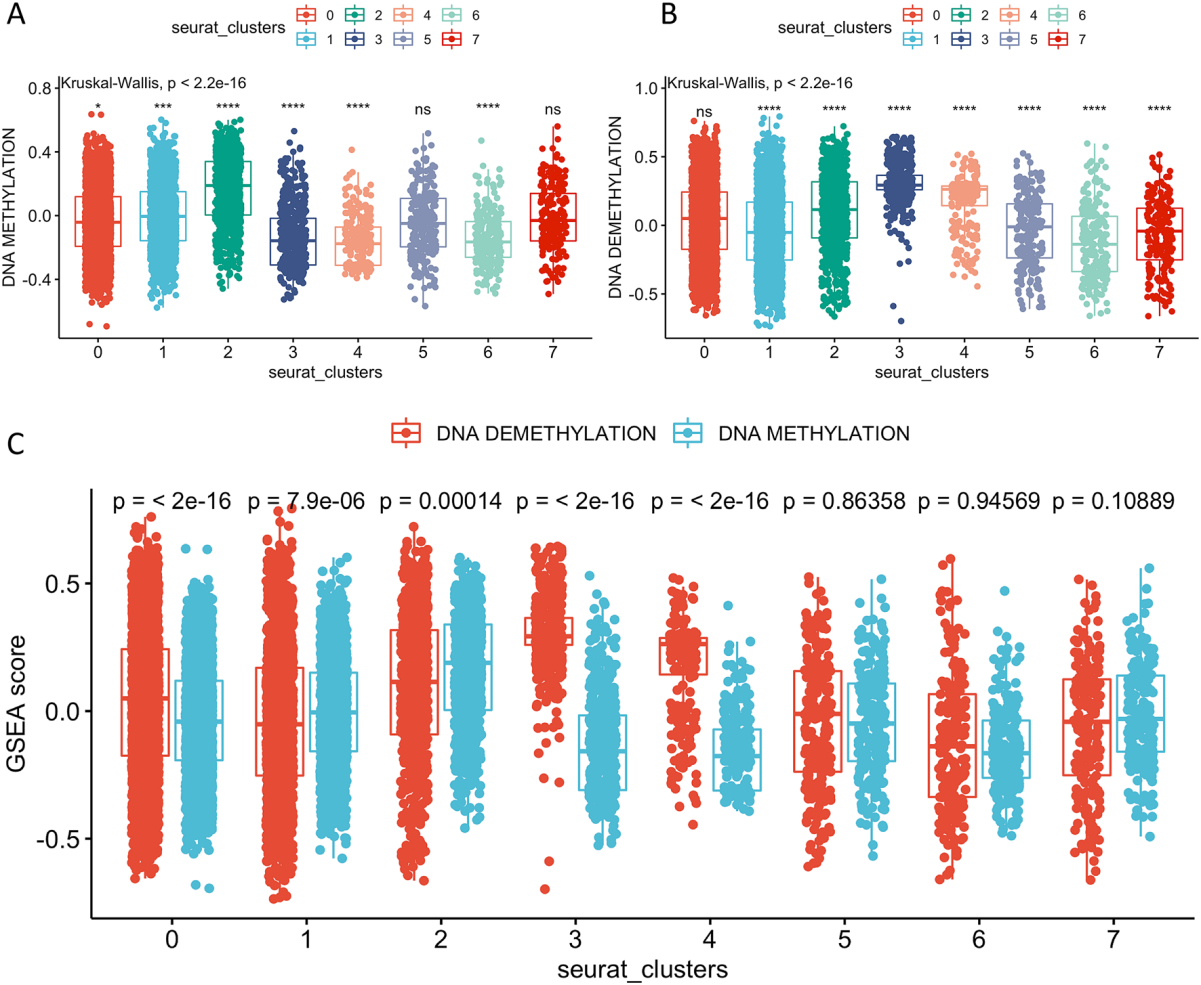
Methylation scores (**A**) and demethylation scores (**B**) for each cluster compared with all the others, and Gene Set Enrichment Analysis scores for methylation and demethylation scores within the same cluster (**C**). Boxplots show medians, interquartile ranges, and outliers. ns: *p* > 0.05; *p ≤ 0.05; **p ≤ 0.01; ***p ≤ 0.001; ****p ≤ 0.0001.

We determined GSVA scores for GO items using GO_DNA_DEMETHYLATION and GO_DNA_METHYLATION for each cell to assess methylation and demethylation activity. Comparison of these scores among all subtypes indicated that Cluster-1 and Cluster-2 had higher methylation scores and Cluster-3, -4, and -6 had lower methylation scores (Fig. 4A). In addition, cluster-2, -3, and -4 had higher demethylation scores and cluster-1, -5, -6, and -7 had lower demethylation scores (Fig. 4B). Our comparison of methylation and demethylation activity within the same cluster indicated that cluster-1 had overall higher methylation activity, due to high methylation scores and low demethylation scores. In contrast, cluster-3 and -4 had overall higher demethylation activity (Fig. 4C). Taken together, this suggested that cells in cluster-1 were mainly hypermethylated and that cells in cluster-3 and -4 were mainly hypomethylated.

**Figure 5:**
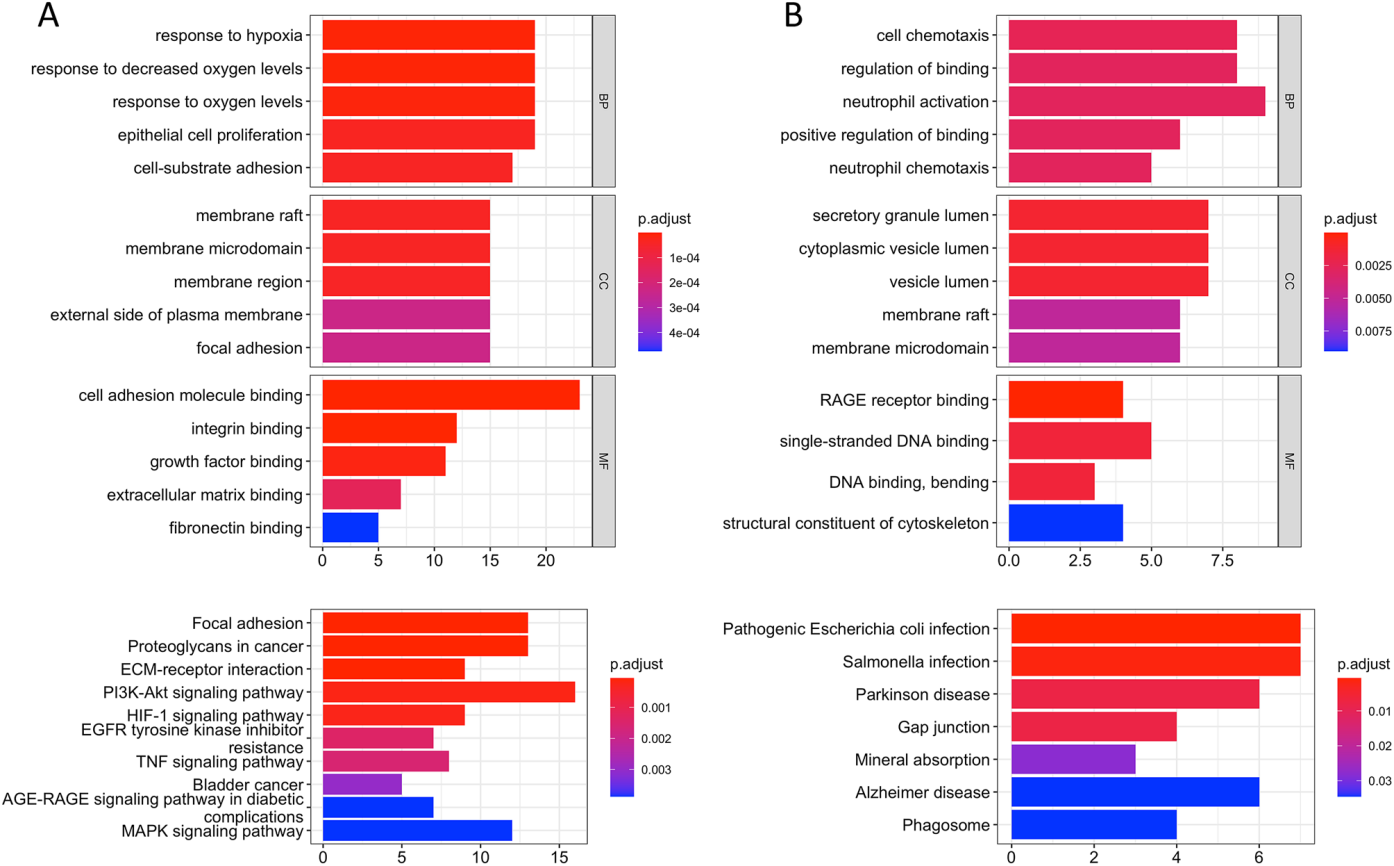
Kyoto Encyclopedia of Genes and Genomes and Gene Ontology enrichment analysis of Cluster-1 (**A**) and Cluster-3 (**B**). *BP* biological process, *CC* cellular components, *MF* molecular functions.

KEGG and GO functional enrichment analysis indicated that hypermethylated cells (cluster-1) were mainly related to the hypoxia response. Notably, genes related to resistance to EGFR tyrosine kinase inhibitors (TKIs) (including *VEGFA*, *ERBB3*, *MET*, *ERBB2*, *KDR*, *STAT3*, *TGFA*) were upregulated in this cluster (Fig. 5A). The hypermethylated cluster-1 and the hypomethylated cluster-3 (Fig. 5B) and cluster-4 (Fig. 3A) were predominantly upregulated in pathways involving focal adhesion and cell junctions.

### Effect of methylation on tumor cell differentiation

**Figure 6:**
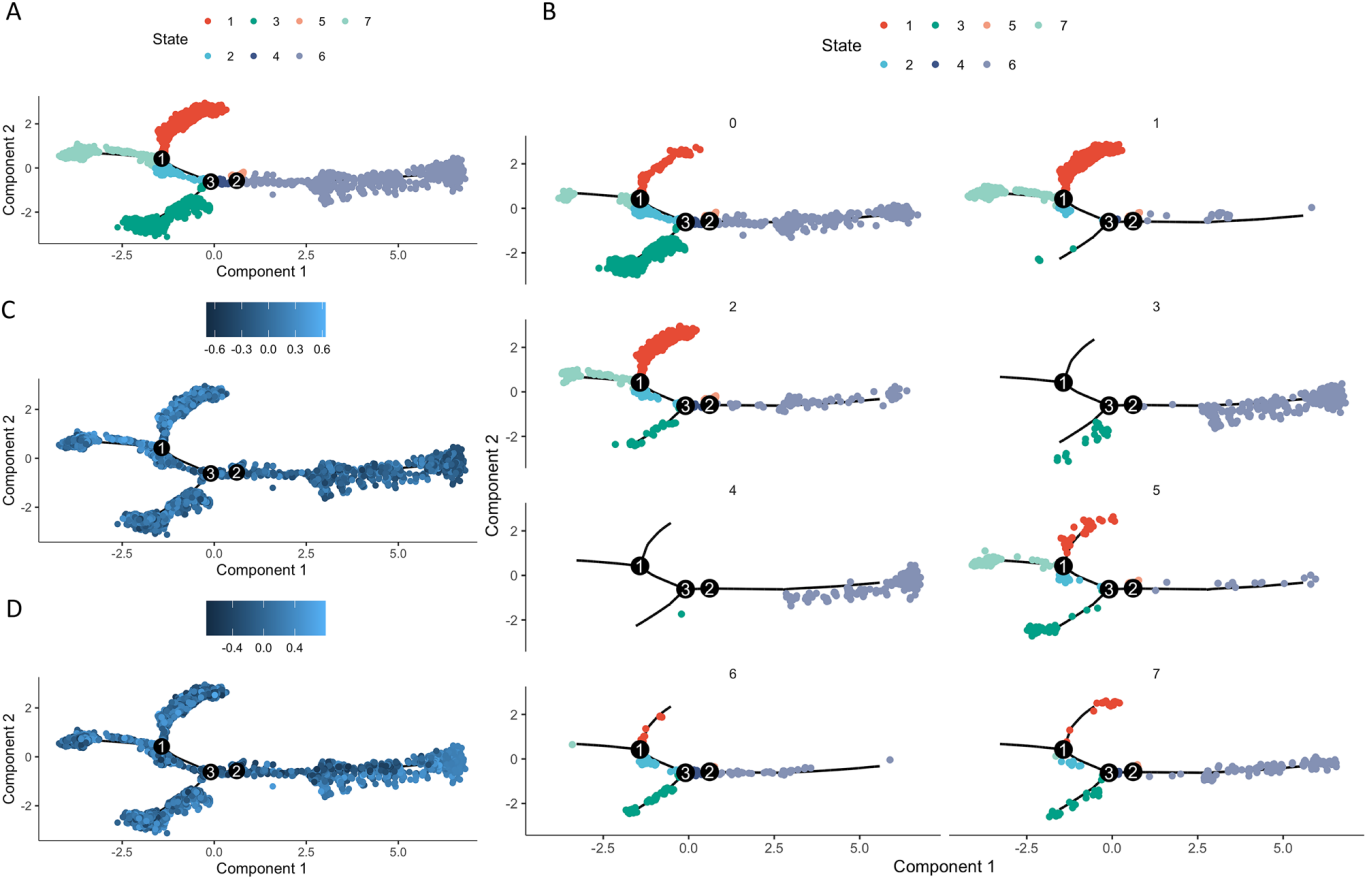
Cell progression pseudotime from Monocle analysis (**A**), methylation scores along the imputed pseudotime (**B**), demethylation scores along imputed pseudotime (**C**), and cell progression pseudotime according to Seurat cluster (**D**).

We applied unsupervised pseudotime inference analysis to investigate the relationship of methylation activity with tumor cell differentiation. Monocle software separated all cells into 7 states (Fig. 6A). Cluster-1 (high methylation activity) was mainly enriched in State-1 and State-7 (Fig. 6B,C). Cluster-3 and Cluster- 4 (low methylation activity) were mainly enriched in State-6 (Fig. 6B,D).

**Figure 7:**
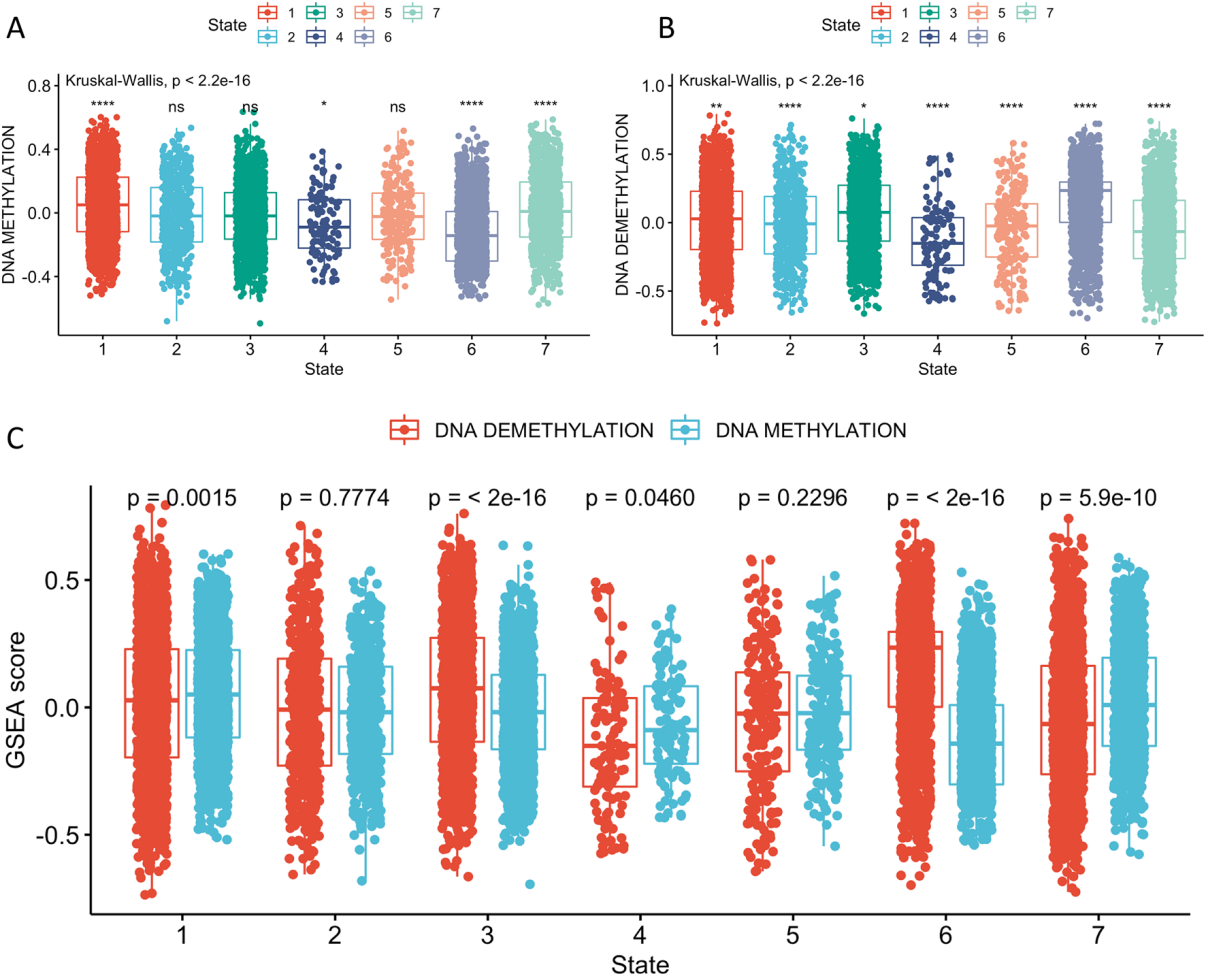
Methylation scores (**A**) and demethylation scores (**B**) for each Monocle state compared with all the other states, and comparison of methylation score and demethylation score within the same state (**C**). Boxplots show medians, interquartile ranges, and outliers. ns: *p* > 0.05; *p ≤ 0.05; **p ≤ 0.01; ***p ≤ 0.001; ****p ≤ 0.0001.

Gene set enrichment analysis (GSEA) scores for the pseudotime states also showed that State-1 and State-7 had significantly higher methylation scores and lower demethylation scores than the other states (Fig. 7A,B). Within individual states, State-1 and State-7 also had significantly higher methylation scores than demethylation scores (Fig. 7C). State-6 had a lower methylation score (Fig. 7A) and higher demethylation score (Fig. 7B) relative to other states, and within the same state (Fig. 7C). Single-cell differentiation pseudotime analysis showed that cell clusters which had more diverse methylation activities were at the ends of the putative pseudotime. This indicates that DNA methylation and demethylation processes were crucial to lung tumor cell progression.

### Association of branch points in pseudotime marking DNA methylation status with developmental decisions

**Figure 8:**
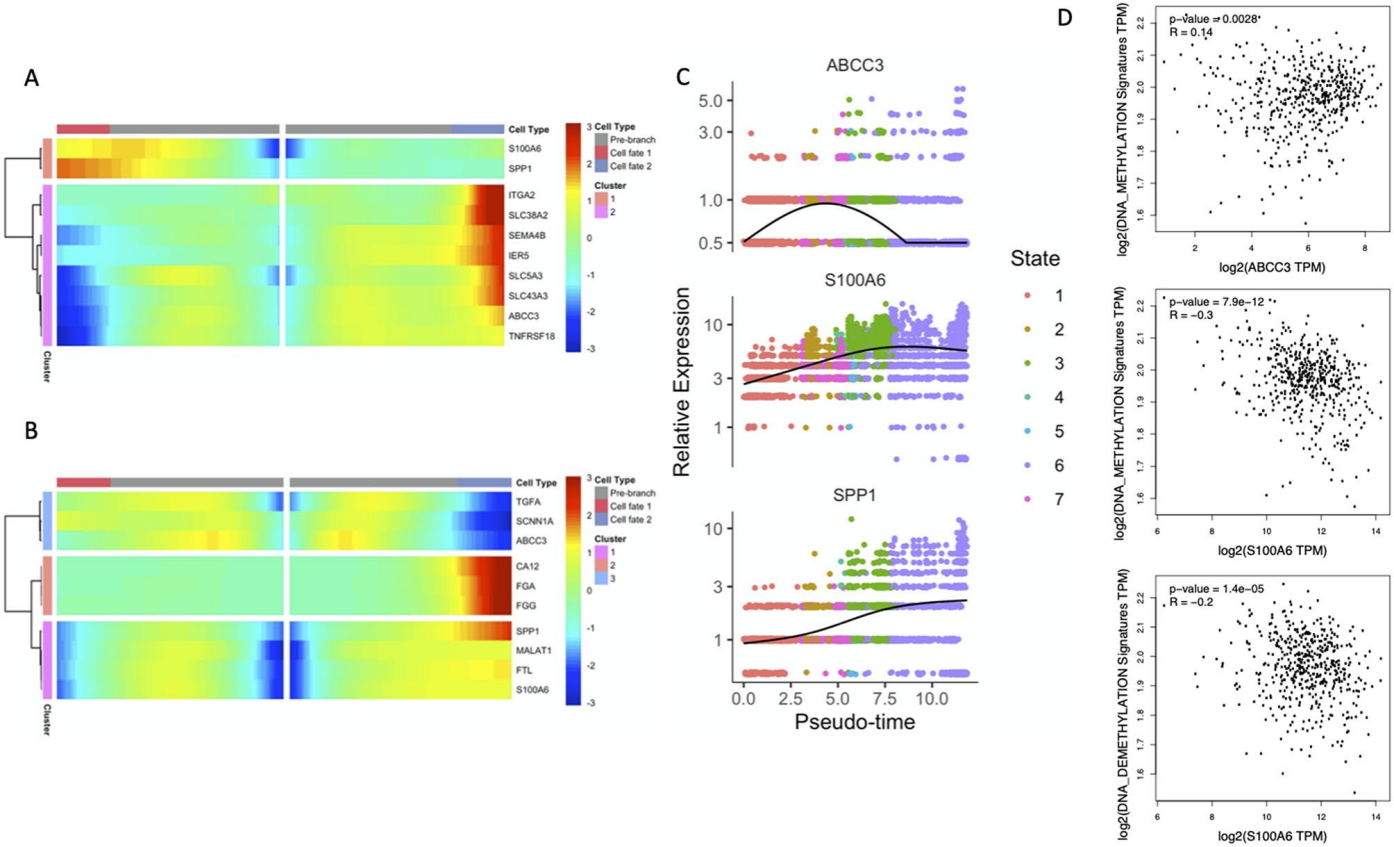
Gene expression profiles (heatmaps) at branchpoint-1 (**A**) and branchpoint-2 (**B**), and expression profiles for *ABCC3*, *SPP1*, and *S1000A6* along the progression pseudotime (**C**). Correlation of the expression of *ABCC3* and *S1000A6* with DNA methylation and de-methylation signatures using the TCGA LUAD datasets (**D**).

Our pseudotime analysis indicated that DNA methylation status affected cell progression, and that branchpoint-1 and -2 were critical turning points for modification of methylation activity and differentiation. During differentiation, progenitor cells undergo early changes that specify the type of terminal cell they will ultimately become. Thus, many progenitor cells can generate different lineages. The above two branchpoints correspond to different changes made early during cell differentiation. Branchpoints mark different transcriptional sub-lineages according to gene expression patterns and help identify key events in different biological processes^6^. To further examine genes affecting cell fate, we analyzed genes that had dramatic changes at these two branchpoints. Heatmaps showed that for branchpoint-1, the *S100A6* and *SPP1* genes were enriched at cell fate-1 (Fig. 8A,C); for branchpoint-2, these same genes were enriched for cell fate-2 (Fig. 8B,C). Both branch points showed that these two genes had higher expression in lung tumor cells with high demethylation activity. In contrast, expression of the *ABCC3* gene was lower in cells with high demethylation activity. We validated these findings using the TCGA LUAD dataset. Correlation analysis of the genes identified above with DNA methylation and demethylation signature scores showed that the expression of *ABCC3* was positively associated with the methylation score, that is hyper-methylation. In contrast, *S100A6* expression was positively correlated with hypo-methylation. These results are in agreement with our scRNAseq results conduced at the bulk RNAseq level (Fig. 8D).

**Figure 9:**
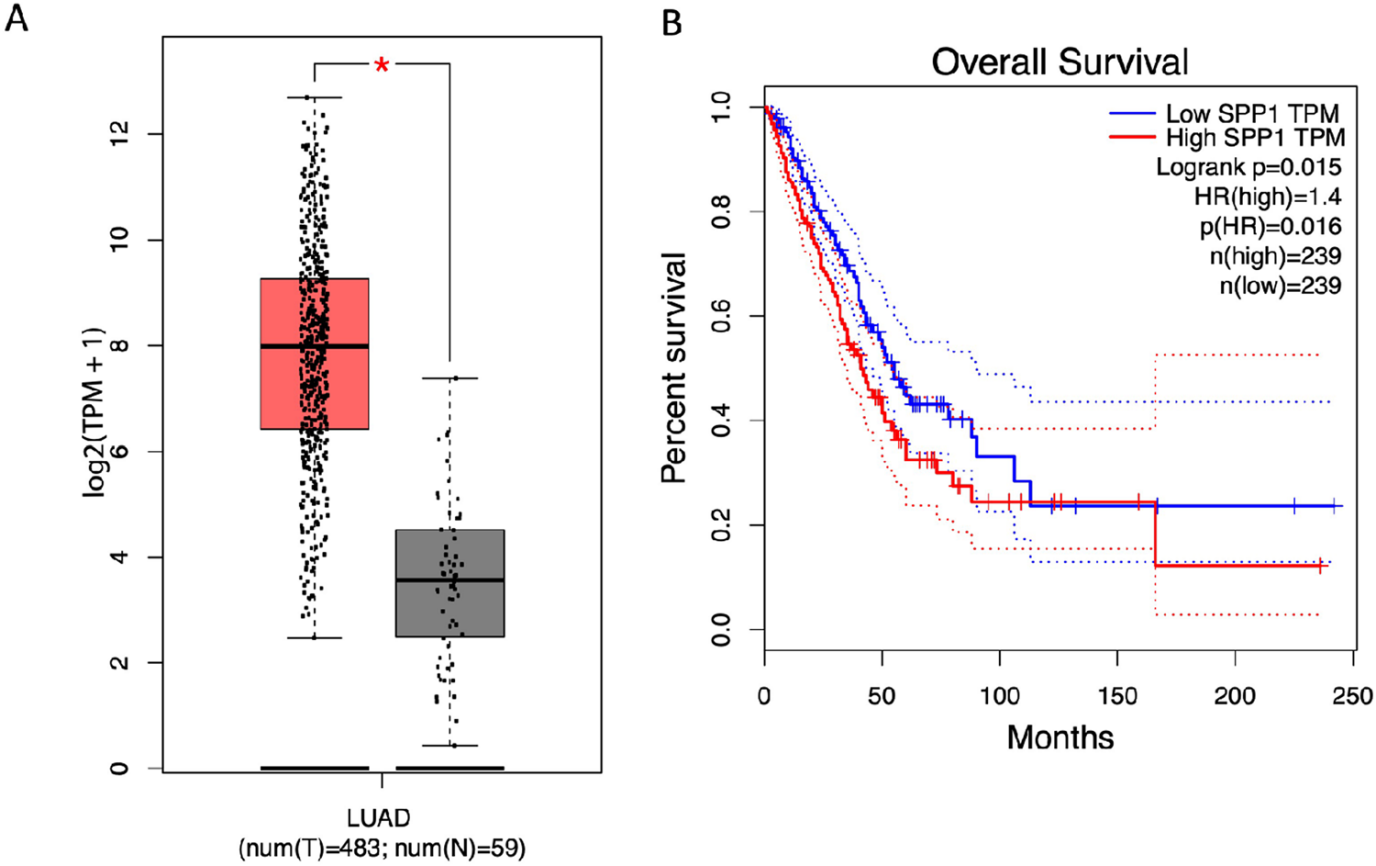
Expression of *SPP1* in normal tissue and tumor tissue from The Cancer Genome Atlas Lung Adenocarcinoma datasets (**A**) and Kaplan–Meier survival analysis for *SPP1* from TCGA data (**B**). Boxplots show medians, interquartile ranges, and total range.

We used TCGA LUAD dataset to examine the prognostic value of these three genes. Bulk RNAseq data from TCGA showed that *SPP1* expression was significantly higher in tumor tissues than normal tissues (Fig. 9A). In agreement, Kaplan–Meier survival analysis showed that lower expression of *SPP1* predicted significantly better outcomes in patients with lung adenocarcinomas (*p* = 0.015, Fig. 9B).

## Discussion

DNA methylation and demethylation are major mechanisms of epigenetic regulation during cell growth and development. DNA methylation is mainly catalyzed by enzymes in the DNA methyltransferase (DNMT) family, including DNMT3A and DNMT3B (which are responsible for de novo methylation) and DNMT1 (which maintains DNA methylation patterns)^7^. In addition, the ten-eleven translocation (TET) enzymes function as 5mC oxidases and also function in DNA demethylation^8^. Hyper-methylation of the promoters of tumor suppressor genes is associated with oncogenesis in many types of cancers^9^. However, tumor cells also exhibit global hypomethylation, and this leads to genomic instability due to genomic rearrangements that disrupt the cell cycle and activation of transposable elements within the genome, leading to further genetic damage^10,11^. Epigenetic changes are among the earliest and most pervasive genomic aberrations during carcinogenesis^12,13^. Thus, markers of DNA methylation may be suitable for the early detection of cancers and as potential therapeutic targets.

Tumor tissues are typically heterogeneous, in that cells within the same tumor often have different gene expression profiles. The newly developed single-cell sequencing protocols make it possible to provide detailed characterization of cell heterogeneity within a tumor^14^. The present study of the expression profiles of lung adenocarcinomas used a single-cell sequencing dataset to profile the methylation and demethylation activity of individual cells. Our results indicated there were clusters of lung tumor cells with remarkably different methylation activities. Our functional enrichment analysis indicated that genes related to focal adhesion had high expression in cells with specific methylation activities. Numerous studies reported that methylation of the promoters of genes that code for cell adhesion proteins increased tumor invasion and progression. For example, Tai et al. reported that increased methylation of the promoter of epithelial cell adhesion molecule (*EpCAM*) was associated with increased expression and proclivity for metastasis^15^. Another study found aberrant methylation of the promoters of three cell adhesion-related genes (*CDH1*, *TSLC1*, and *TIMP3*) in NSCLC was associated with more severe clinicopathology of the tumor and exposure to various environmental risk factors^16^.

However, most previous studies examined the methylation levels of selected gene sets and bulk tumor tissues. We identified the relationships of precise tumor cell subclusters with abnormal invasive and metastatic potentials, and used single-cell GSEA analysis to examine global changes in DNA methylation status. Our results verified that changes in DNA methylation impacted tumor progression, especially invasion and metastasis. In particular, tumor cell clusters with high methylation activity also had a significant hypoxia response. Thienpont et al.^17^ found that tumor hypoxia reduced the activity of TET, a crucial DNA demethylation enzyme, and consequently increased the hypermethylation of specific gene promoters. They also found that hypoxic tumor tissues had hypermethylation of the promoters of tumor suppressor genes, and that restoration of tumor oxygenation abrogated the hypermethylation in mouse breast cancers^17^. However, another study reported that DNA methylation affected the accessibility of the HIF transcriptional factor to its binding site; DNA hypomethylation exposed HIF binding sites and induced HIF-dependent expression of cryptic unstable transcripts (CUTs). Tumors with high immune checkpoint expression also had decreased DNA methylation and higher expression of CUTs. These results indicated that the interplay between hypoxia and DNA methylation might influence tumor immunotolerance^18^.

Our identification of tumor cell subtypes using single-cell analysis supports this interpretation. Thus, an in-depth analysis of these clusters may provide a better understanding of the mechanism of immunotolerance and its regulation by hypoxia and DNA-methylation, and may also provide a basis for novel cancer immunotherapies. We also found significantly higher expression of genes that were related to resistance to EGFR-TKIs. TKIs have widespread clinical applications in cancer, but drug resistance greatly limits their efficacy^19^. Our finding that EGFR-TKI resistance was associated with overall hypermethylation activity suggested that DNMT inhibitors, such as azacytidine and decitabine, may help to relieve resistance to EGFR-TKIs.

Our pseudotime analysis of lung tumor cells indicated that hyper- and hypomethylated clusters were mainly at different ends of the progression timeline, suggesting an important role of methylation status during lung adenocarcinoma progression. Our examination of genes whose expression may determine cell fate indicated that *S100A6* and *SPP1* had higher expression in globally hypomethylated cells, and that *ABCC3* had the opposite pattern (Fig. 1). The expression profiles of these genes determined the pseudotime of cell differentiation at the most important two branch points related to DNA methylation. Recent studies reported that *SPP1* was associated with cell growth and invasion during tumorigenesis and metastasis^20^; *SPP1* was overexpressed in cancers of the lung^21^, colon^22^, breast^23^, and prostate^24^; and *SPP1* expression correlated with tumor stage and aggressiveness. *S100A6* functions in the regulation of cell cycle progression and differentiation. Aberrant expression of *S100A6* was previously reported in cancers of the pancreas^25^, colorectum^26^, gastric system^27^, and breast^28^. A study of S100 proteins concluded that they may promote cancer progression by altering pathways related to cell survival and apoptosis^29^. More specifically, several studies reported that increased expression of *S100A6* promoted cell proliferation by regulating the expression of *IL‑8*, *CDK5*, *CDK4*, *MCM7*, *Bcl2*, and could be used as a marker of tumor aggressiveness in gastric cancers^27,30^.

Previous epigenetic studies emphasized the relationships between methylation of the promoters of *SSP1* and *S100A6* and expression of these genes, but few studies examined the relationship of *SPP1* and *S100A6* expression with global methylation levels of tumor cells. Hypomethylation of highly repeated DNA sequences is more common in cancer tissues than normal tissues^31^, and global hypomethylation often occurs at the very beginning of tumorigenesis^32,33^. Although there are variations among different types of tumors, global hypomethylation is generally related to increased cancer progression and malignancy^34–36^. Although global DNA hypomethylation is tightly linked to the formation of repressed chromatin domains, and does not occur in the presence of histones H3K9me3 or H3K27me3, the mechanisms that drive these alterations are still uncertain^37^. Our single-cell analysis indicated that expression of *SPP1* and *S100A6* were associated with global hypomethylation of lung adenocarcinoma cells. This result implicates these two genes in the regulation of the global methylation level of lung adenocarcinoma cells.

*ABCC3* has greater expression in many cancers and is also a marker of multidrug resistance. Higher expression of *ABCC3* correlated with lymph node involvement, advanced TNM stage, more malignant histological type, multiple-resistance to anti-cancer drugs, and reduced overall survival in NSCLC^38,39^. Our analysis showed that the expression of *ABCC3* was positively correlated with global DNA hypermethylation of lung cancer cells.

Recent advances in next-generation sequencing and single-cell technologies allowed the examination of cell heterogeneity within tumors. We used these methods to identify different cell profiles based on clustering of similar cells into subgroups, and then compare the gene expression patterns of different subgroups. Our single-cell sequencing methods thus provided a detailed classification of lung adenocarcinoma cells, identification of clusters based on global methylation profiles, and examination of the functions of these clusters. Our pseudotime analysis suggested that global methylation level affected the differentiation of tumor cells and that *SPP1* was associated with methylation level and patient prognosis.

## Methods

### Data sources

RNA-seq datasets were from The Cancer Genome Atlas (TCGA) and clinical data for lung adenocarcinoma were from the University of California Santa Cruz (UCSC) Xena browser (https://xenabrowser.net/). Single-cell RNA sequencing datasets for lung tumors were downloaded from ArrayExpress (https://www.ebi.ac.uk/arrayexpress/) with the accession numbers E-MTAB-6149 and E-MTAB-6653 (Table 1). Table 2 summarizes the clinical data of these three patients. All methods were carried out in accordance with relevant guidelines and regulations.

**Table 1:** Samples used for data analysis (E-MTAB-6149 and E-MTAB-6653).

Sample	Patient #	Tumor site	Cells (N)
BT1290	3	Border	497
BT1291	3	Middle	842
BT1292	3	Core	1104
BT1295	4	Border	321
BT1296	4	Middle	859
BT1297	4	Core	1520
BT1375	6	Core	346
BT1376	6	Middle	528
BT1377	6	Border	234

**Table 2:** Characteristics of the 3 NSCLC patients included in this study.

Patient #	Age, years	TNM	Stage	Carcinoma type	Affected lobe	Smoking status
3	68	pT4N2M0	IIIB	Adenomatous	Right upper	Former
4	64	pT2aN1M0	IIB	Adenomatous	Left upper	Former
6	65	pT4N1M0	IIIA	Adenomatous	Left upper	Former

### Single-cell RNA-seq data acquisition and pre-processing

Raw gene expression matrices for each sample were analyzed using the Seurat package (ver. 3.2.2) for R software^40^. Count matrices were filtered again by removing cell barcodes that had fewer than 201 genes. The remaining cells were first integrated using canonical correlation analysis (CCA) for the 5000 genes with the greatest variation in expression. All variably expressed genes were used to construct principal components (PCs), and the 30 PCs with the greatest variance in the dataset were selected. Clusters were calculated using the FindClusters function with a resolution of 0.2, and were visualized using the t-distributed stochastic neighbor embedding (t-SNE) for dimensional reduction. Up-regulated marker genes in each cluster were calculated using the FindAllMarkers function with only.pos = TRUE and an adjusted p-value below 0.05.

### Functional enrichment analysis

GO and Kyoto Encyclopedia of Genes and Genomes (KEGG) enrichment analysis for significantly up-regulated genes were analyzed using the ClusterProfiler^41^ package for R software. The 5 GO terms with the smallest adjusted *p* values for molecular function (MF), biological process (BP), and cellular components (CC), and the top 10 enriched KEGG pathways were shown.

### Estimation of DNA methylation and de-methylation activity

To evaluate DNA methylation and de-methylation activity (i.e. the global methylation level of each cell), a GSVA score was calculated for each cell on GO items with GO_DNA_DEMETHYLATION (GO:0080111) and GO_DNA_METHYLATION (GO:0006306) using the GSVA package for R software^42^.

### Monocle analysis

The Monocle package (version 2.16.0) for R software was used to plot pseudotime to illustrate the behavioral similarity and transitions of lung tumor cells^6^. The integrated expression matrix derived from Seurat was used to build a CellDataSet for the Monocle pipeline. All cells were finally aggregated into 7 different states according to the pseudotime inferred from the expression profiles.

### Survival analysis and Expression analysis for bulk RNAseq data

Survival analysis and comparison of expression in tumor and normal tissues from TCGA lung adenocarcinoma (LUAD) dataset was performed using GEPIA (http://gepia.cancer-pku.cn/)^43^. Group cutoff of survival analysis was set as the median expression value of each testing gene.

## Data Availability

The datasets generated and analyzed during the current study are available from the corresponding author on reasonable request.
